# Reassurance on false negatives in the Manchester COVID19 Urgent Eyecare Service (CUES)

**DOI:** 10.1038/s41433-021-01774-w

**Published:** 2021-09-28

**Authors:** Emma Williams, Wendy Craven, Helen Wilson, Felipe Dhawahir-Scala, Matthew Jinkinson, William D. Newman, Robert A. Harper

**Affiliations:** 1 Manchester Royal Eye Hospital and Manchester Academic Health Sciences Centre, Manchester University NHS Foundation Trust Manchester, Manchester, UK; 2 Primary Eyecare Service, 2.3 Waulk Mill, Manchester, UK; 3 Greater Manchester Eye Health Network, Health & Social Care Partnership, Manchester, UK; 4 Division of Pharmacy and Optometry, School of Health Sciences, Faculty of Biology, Medicine and Health, University of Manchester, Manchester, UK

**Keywords:** Eye diseases, Health services

In June 2020 we described the development and implementation of COVID19 Urgent Eyecare Service (CUES) in Greater Manchester [1]. We subsequently published an evaluation of the service [2], demonstrating a high level of primary care activity, an approximate ~14% referral rate into secondary care, and reduced footfall into the hospital’s emergency eye department (EED). Furthermore, the case mix of patients attending EED appeared to be more complex than that seen prior to introducing CUES, with primary care managing lower risk urgent eyecare presentations; however, one limitation of our evaluation was an acknowledged absence of examination for possible false negatives within CUES. Indeed, in primary eyecare services in general, there has been a paucity of evaluation of the potential for false negatives, although some studies have examined this issue in glaucoma referral filtering services [3–6] and one study did assess clinical safety in a Minor Eye Conditions Service (MECS) [7], a service more closely aligned to CUES in accommodating urgent eyecare in the community. In this article, we present an analysis tracking a large population of cases seen in primary care CUES in Manchester where we specifically monitored the potential for *non-referred* cases to attend the hospital’s EED within 28 days (a proxy for potential missed urgent eyecare cases, i.e. false negatives).

**Fig. 1:**
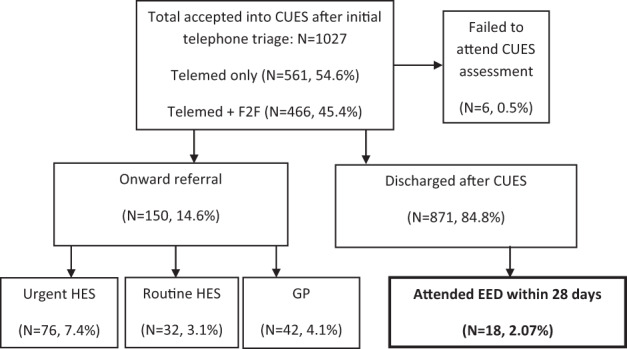
Flowchart for primary care CUES cases in Manchester and their subsequent management. The proportion of potential false-negative cases we consider is seen within the context of all non-referred CUES episodes (871) versus the total number of episodes seen within the service in the evaluation timeline.

A total of 1027 patient episodes deemed suitable for CUES following initial telephone screening in August, September and October 2020 were subsequently reviewed. Patient episodes rather than patient numbers were considered, since a small proportion of assessments (*N* = 42) were noted to be follow-up assessments carried out for the same patient. The flow diagram in Fig. 1 shows the outcome of the assessments from all episodes. Of 871 episodes resulting in discharge from CUES without referral, a total of 18 patients were identified as having attended Manchester Royal Eye Hospital’s (MREH) EED within the following 28 days, giving a *potential* false-negative rate of 2.07% (95% confidence interval 1.23–3.25%). In order to establish if these cases were genuine false negatives and to examine potential reasons why these patients presented to EED following a CUES discharge, we retrospectively examined the EED records, cross-referencing this information with primary care CUES records (while acknowledging that these provide a summary of optometric consultations in CUES versus any separately held practice practitioner records). This process allowed comparison between diagnostic and management decisions made in CUES and in EED. The records were reviewed by two EED hospital optometrists (E.W. and H.W.) with EED’s consultant head of acute services (F.D.S.) who risk rated each case by consensus into the following categories: A - Agreement on diagnosis/management: No risk to patient; B - Disagreement on diagnosis/management: No/low risk of threat to sight; and C - Disagreement on diagnosis/management: Moderate/high risk of threat to sight. Table 1 summarises comparative data for CUES and EED diagnoses and management decisions with companion risk ratings.

**Table 1:** Comparison between the CUES (Manchester COVID19 Urgent Eyecare Service) and EED (emergency eye department) diagnoses and management decisions, along with HES clinicians’ risk assessment, graded as follows: A - Agreement on diagnosis/management: No risk to patient; B - Disagreement on diagnosis/management: No/low risk of threat to sight; and C - Disagreement on diagnosis/management: Moderate/high risk of threat to sight.

Case number	CUES assessment type	CUES diagnosis and management	EED diagnosis and management	Time (days) between CUES and EED	Risk grading
1	Telemed and F2F	Flare up of uveitis, patient using old bottle of maxitrol for 7 days. Appears to have settled. Advised not to use old drops and return if symptoms worsen.	Recurrent uveitis associated with HLA-B27. Known steroid responder. Treated with steroids, mydriasis and tiopex.	3	A
2	Telemed and F2F	PVD. Discharged with advice.	PVD. Discharged with advice.	6	A
3	Telemed	Viral conjunctivitis. Advised to contact practice if no improvement in 1 week.	Episcleritis and dry eye treated with mild steroid and lubricants.	5	B
4	Telemed	Bacterial conjunctivitis. Advised chloramphenicol and lubricants.	Known complex glaucoma patient on triple therapy, with significantly elevated IOPs. Added iopidine and diamox, review 1/52.	5	C
5	Telemed	Viral conjunctivitis. Hygiene advice and SOS.	Viral conjunctivitis, pseudomembrane removed, CPL and lubricants. SOS.	7	A
6	Telemed	Migraine. Discharge with advice.	Likely migraine. BP and basic bloods normal. Discharged.	2	A
7	Telemed and F2F	PVD. Discharge with advice.	PVD. Discharge with advice.	10	A
8	Telemed and F2F	Ectropion, blepharitis, dry eye. Lid hygiene, lubricants, option of surgery discussed but declined. SOS.	Severe dry eye secondary to blepharitis. CPL, lid hygiene and lubricants.	6	A
9	Telemed and F2F	PVD, discharge with advice.	PVD with retinal haemorrhage, observe.	0	A
10	Telemed and F2F	PVD, discharge with advice.	Dry eye secondary to CL wear, PVD.	21	A
11	Telemed and F2F	Corneal abrasion, lubricants, return if no improvement.	Corneal abrasion, CPL and lubricants, discharge.	1	A
12	Telemed	Bacterial conjunctivitis, CPL and hygiene.	Mild allergic eye disease, opatanol and lubricants, SOS.	5	B
13	Telemed and F2F	Floaters, no sign of PVD or RD.	3rd nerve palsy (2 days history). Blood tests, MRI, review in neuro-ophthalmology.	5	A
14	Telemed	Chalazion, warm compresses and massage, SOS.	Asked to review by paediatricians’ team to exclude disc swelling due to history of headache, nausea and vomiting. Normal ocular exam.	20	A
15	Telemed	Lid infection. Cleaning and warm compresses.	Viral keratoconjunctivitis. Mild steroid, CPL, lubricants and sodium cromoglicate.	27	B
16	Telemed and F2F	PVD, discharge with advice	PVD, discharge with advice.	16	A
17	Telemed	Bacterial conjunctivitis, CPL	Recurrent HLA-B27 associated anterior uveitis and non-necrotizing anterior scleritis. Steroids, cycloplegia, flurbiprofen and omeprazole. Follow up arranged.	2	C
18	Telemed and F2F	Bacterial conjunctivitis. Lubricants and hygiene.	Conjunctivitis. Old retinal tears noted as incidental finding - retinopexy.	10	A

*Telemed* telemedicine, *F2F* face to face, *PVD* posterior vitreous detachment, *CPL* chloramphenicol, *SOS* see on symptoms, *CL* contact lens, *IOP* intraocular pressure, *RD* retinal Detachment, *HES* hospital eye service.

Reassuringly, in 13 cases there were no concerns over the management decisions taken by the CUES optometrist. In three cases there was disagreement on diagnosis and management, although the disagreement posed a low level of risk to the patient’s sight. All three cases were red eye or eye infection dealt with by telemedicine only, following a protocol created for primary care optometrists relevant at the material time, limiting face-to-face assessment of red eye during the pandemic to conditions that were potentially sight-threatening, albeit relying on a high-quality history and symptoms assessment to identify “red flags”.

The remaining two cases, in which the disagreement on diagnosis and management was classified as ‘C’ (i.e. moderate-to-high risk of threat to sight), represented 0.23% (95% confidence interval 0.03–0.83%) of all non-referred cases. These cases are considered further: Case 4 (Table 1) involved a 56-year old female patient assessed by telemedicine, noted to have a red right eye with sticky discharge. The vision in the right eye was noted to be worse and the left was stable. After CUES assessment the patients was diagnosed with bacterial conjunctivitis and treatment with chloramphenicol and lubricants was advised. Although the patient subsequently reattended CUES for a face-to-face assessment and was referred, her referral was routine, and she attended EED herself complaining of a “pressure sensation” within the eyes and was found to have intra-ocular pressures of 31 mmHg bilaterally with very narrow angles due to bilateral peripheral anterior synechiae. The patient had a history of poorly controlled narrow angle glaucoma, treated with peripheral iridotomies, bilateral cataract surgery, and triple topical glaucoma therapy. Previously, following her right cataract procedure in 2019, she had attended EED for emergency treatment. Since her more recent EED episode post-CUES, she has undergone glaucoma surgery. In CUES, her complex glaucoma history did not appear to have been elucidated. Case 17 (Table 1) involved a 45-year old female diagnosed with bacterial conjunctivitis in CUES and treated with chloramphenicol for a red, painful, watery left eye. The patient attended EED 2 days later, where a history of recurrent acute anterior uveitis in association with ankylosing spondylitis was noted at triage. She was found to have a recurrence of uveitis with non-necrotizing anterior scleritis and was treated accordingly. A full history and symptoms assessment, including previous eye problems and general health status, would have alerted the CUES practitioner to the more likely diagnosis of uveitis.

In conclusion, this evaluation of a non-referred population seen in primary care CUES supports the view that the service is clinically safe. The false-negative rate of 0.23% for moderate-to-high risk of sight loss cases in the cohort reviewed is reassuringly low within the context of: first, the pandemic and emphasis on telemedicine at the material time; second, CUES evolving to accommodate a higher proportion of face-to-face assessments and with the potential for further guidance for participating optometrists about the importance of a thorough history and symptoms evaluation; and finally, CUES comparing favourably with currently commissioned primary care services where false-negative rate evidence is available [3–7]. We believe that this additional analysis and our earlier evaluation strongly supports the ongoing commissioning of CUES in primary care.
